# Characterization of Carboxylated Cellulose Nanocrystals Isolated Through Oxalic Acid Hydrolysis from Solid Residues of Softwood-Derived Glycol Lignin Production †

**DOI:** 10.3390/molecules30142922

**Published:** 2025-07-10

**Authors:** Thi Thi Nge, Tatsuhiko Yamada

**Affiliations:** Center for Advanced Materials, Forestry and Forest Products Research Institute (FFPRI), 1 Matsunosato, Tsukuba 305-8687, Japan; yamadat@nethome.ne.jp

**Keywords:** carboxylated cellulose nanocrystals, recyclable oxalic acid, softwood, solid residues, glycol lignin

## Abstract

The efficient use of renewable lignocellulosic biomass has attracted wide interest, as it promises to reduce the environmental impact of fossil fuel consumption. A recently developed batch-scale process, which produces glycol lignin (GL) from softwood biomass, generates a considerable amount of cellulose-rich solid residues (SRs) as a byproduct. In this study, usable cellulose was isolated from SRs in the form of carboxylated cellulose nanocrystals (O-CNCs). The properties of O-CNCs were investigated to establish a possible integrated biomass utilization system based on the GL production technology. Three different forms of purified SRs—never-dried (N-Cel), freeze-dried (F-Cel), and vacuum-dried (V-Cel) cellulose—were subjected to oxalic acid (OA) hydrolysis at 95 °C for 4 h. The average length of O-CNCs ranged from 90 to 120 nm and the height ranged from 3 to 6 nm for separate particles and from 8 to 20 nm for aggregates. The carboxyl group content was 0.11–0.23 mmol/g O-CNCs. The overall results indicated that the yields, dimensions, surface charges, and thermal stability of the O-CNCs were largely influenced by the nature of the starting cellulose. In addition, O-CNCs prepared from recycled OA exhibited similar properties to those prepared from fresh OA.

## 1. Introduction

The efficient use of renewable natural resources is expected to mitigate climate change and avert the looming depletion of fossil fuels [1,2]. Cellulose is among the most abundant organic biopolymers on Earth, constituting 40–60% of the cell wall components of vascular plants. Lignin, another major constituent (15–35%) of cell wall components is the most abundant aromatic biopolymer and a suitable sustainable carbon source for fossil fuel replacement [3,4]. Therefore, researchers are seeking economically viable technological developments and sustainable practices for lignocellulosic biorefineries setups. In particular, the efficient bioconversion of all three major constituents of lignocellulosic biomass (cellulose, hemicellulose, and lignin) to value-added products, biochemicals, and biofuels is gaining global attention [1,2].

A recently developed batch-scale production system converts softwood biomass to polyethylene glycol (PEG)-modified glycol lignin (GL), a high-quality lignin derivative with substantially controlled chemical structures and thermal properties [5,6]. The GL production test plant installed at the Forestry and Forest Products Research Institute (FFPRI) is operated with 50 kg wood meal per batch. A simple processing system directly extracts the lignin derivatives from local biomass (Japanese Cedar, JC) through acid-catalyzed PEG solvolysis at 140 °C for 90 min under atmospheric pressure. The physicochemical and thermal properties of the PEG-modified GLs can be varied by adjusting the size distributions of the JC wood meal particles and the molecular mass of liquid PEG. The JC particles with average meal sizes of 1.6 mm, 0.8 mm, and 0.4 mm are called JC-L, JC-M, and JC-S, respectively, and the PEGs with molecular masses of 600, 400, and 200 are called PEG600, PEG400, and PEG200, respectively [5]. Although GL-containing high performance and functional materials have been successfully fabricated from GLs [7,8], the process yields a considerable amount of cellulose-rich solid residues (SRs) as a byproduct. The main product, GLs is considered a reliable, quality feedstock in a wide range of industrial applications, but characterizing the SRs might pave the way for integrated biomass utilization systems based on GL production technology. Therefore, the present study aims to isolate usable cellulose from SRs in the form of cellulose nanocrystals (CNCs) [9].

Typical CNCs are rod-shaped (whisker-shaped) nanomaterials with lengths and widths of 3–50 nm and 50–500 nm, respectively, which retain the inherent properties of cellulose [10]. With their unique physicochemical and optical properties, CNCs isolated from biosources, such as wood, cotton, and straw have emerged as attractive functional components in the production of various renewable bio-based materials, such as supercapacitors [11], anti-counterfeiting [12], optical films with controllable structural color [13], and colorimetric responsive films [14], which are demanded in sustainable future societies. Apart from pure cellulose sources such as bleached wood pulp and cotton, initial raw cellulose materials are usually pretreated with mechanical, chemical, or enzymatic methods, or a combination of these [15]. CNCs are then isolated through a preparation method such as mineral acid, solid acid, or organic acid hydrolysis, enzymatic hydrolysis, oxidative degradation, subcritical water hydrolysis, or treatment with ionic liquids and deep eutectic solvents (DESs) [15,16].

However, CNCs are mainly produced through the controlled acid hydrolysis of cellulose using concentrated mineral acids [10,16], particularly sulfuric acid [17,18,19] and hydrochloric acid [20]. CNC preparation also uses phosphoric acid [21,22], phosphotungstic acid [23], and hydrobromic acid [24]. The morphology, physical dimensions, surface charge, and yield of the resulting CNCs depend on the cellulose source, acid concentration, reaction time, and reaction temperature. CNCs produced through concentrated sulfuric acid hydrolysis (S-CNCs) exhibit excellent colloidal stability conferred by negatively charged half-ester sulfate groups on their surfaces [10,17,18,19]. Therefore, most studies on the lyotropic liquid crystalline (nematic and chiral nematic) properties of CNCs for photonics and other specialty applications are based on S-CNCs [11,12,14]. However, the introduction of charged sulfate groups compromises the thermal stability of the S-CNCs. In addition, the high cost, difficulty of recovering the acid, and difficulty of disposing of the large amounts of salts from neutralization are concerns mainly in large-scale production using concentrated sulfuric acid hydrolysis [25].

Regardless of the employed hydrolytic acid, the CNC surface possesses abundant hydroxyl (–OH) groups, enabling a diverse range of chemical functionalizations such as esterification, etherification, oxidation, and silylation to fulfill the desired material properties. CNCs have been physically and chemically modified to (1) introduce stable negative or positive electrostatic surface charges that improve their dispersion stability, (2) tune their surface energy characteristics to enhance their compatibility, especially when used in conjunction with nonpolar or hydrophobic matrices in nanocomposites, without degrading their original crystal morphology [10,16]. Surface carboxylation is a common approach for improving the dispersion stability of CNCs in various organic solvents and enhancing the compatibility between CNCs and various nonpolar matrices. Carboxylated CNCs with varying physical dimensions and carboxyl group contents have been obtained through 2,2,6,6-tetramethylpiperidine-1-oxyl (TEMPO)-mediated oxidation [26], ammonium persulfate (APS) oxidation [27,28], periodate–chlorite oxidation [29], and potassium permanganate oxidation [30]. However, these methods are disadvantaged by a high chemical cost, large energy consumption, and limited reagent recyclability and reusability.

Solid organic dicarboxylic acids such as oxalic acid and maleic acid are suitable alternatives to concentrated mineral acids because they have low corrosivity, easy recyclability, and enable a simple one-step hydrolysis reaction for carboxylated CNC preparation [25,31,32,33,34]. The oxalic acid (OA) hydrolysis of cellulose involves two concurrent acid-catalyzed reactions: the hydrolysis of amorphous cellulose segments, and esterification to introduce carboxylic acid functionality on the isolated CNC surfaces [25]. Most studies on O-CNC preparation through OA hydrolysis have utilized cotton [31,33], hardwood bleached kraft eucalyptus pulp (BEP) [25,31,32] and softwood dissolving pulp (SDP) [31]. To our knowledge, OA hydrolysis for CNC production from softwood-derived SRs has not been reported, but is worth investigating from the sustainability and circular economy perspectives. In this study, carboxylated O-CNCs were isolated through the OA hydrolysis of SRs-derived cellulose with three different forms: never-dried (N-Cel), freeze-dried (F-Cel), and vacuum-dried (V-Cel) cellulose. The effects of the nature of the starting cellulose material on the yields, physical dimensions (morphology), surface charges, carboxyl group contents, and thermal stabilities of the resulting O-CNCs are studied and the reusability of OA is assessed.

## 2. Results and Discussion

### 2.1. Pretreatment of As-Received Solid Residues (SRs)

**Figure 1:**
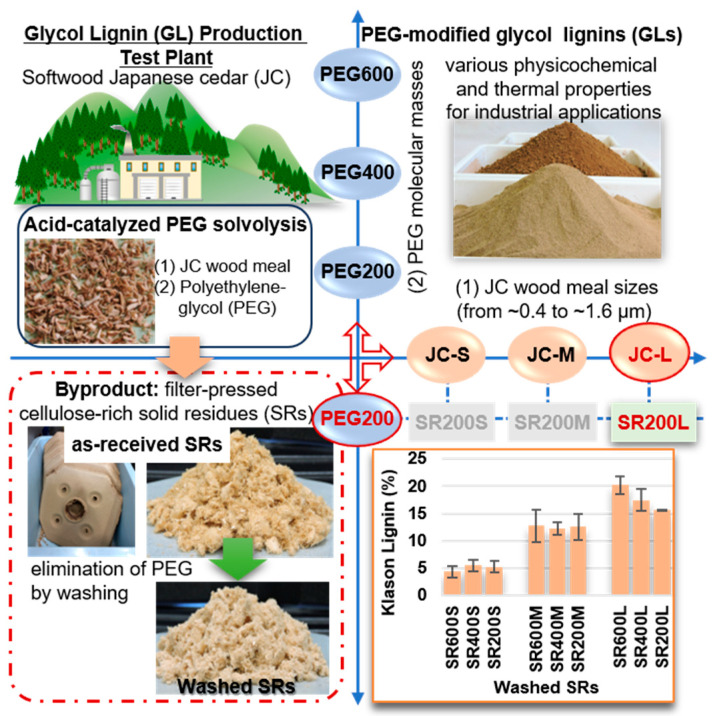
Flow diagram of glycol lignin (GL) production from softwood biomass. Cellulose-rich solid residues (SRs) are obtained as a byproduct.

GL production from JC with three different wood meal sizes (JC-L (~1.6 mm) > JC-M (0.8 mm) > JS-S (~0.4 mm)) and PEG with three different molecular masses (PEG600 > PEG400 > PEG200) [5] yielded nine filter-pressed SR samples (see Figure 1). The unreacted PEG and a certain amount of lignin remaining in the as-received SRs were eliminated by washing with water and applying two successive purification (bleaching) processes. After three successive washings, the dried weight of the washed SRs was approximately one third of the initial wet weight of the as-received SRs. Next, the Klason lignin content (Figure 1) of the washed SRs was analyzed, suggesting that increasing the wood meal size and PEG molecular mass in the solvolysis reaction increased the residual lignin content.

**Figure 2:**
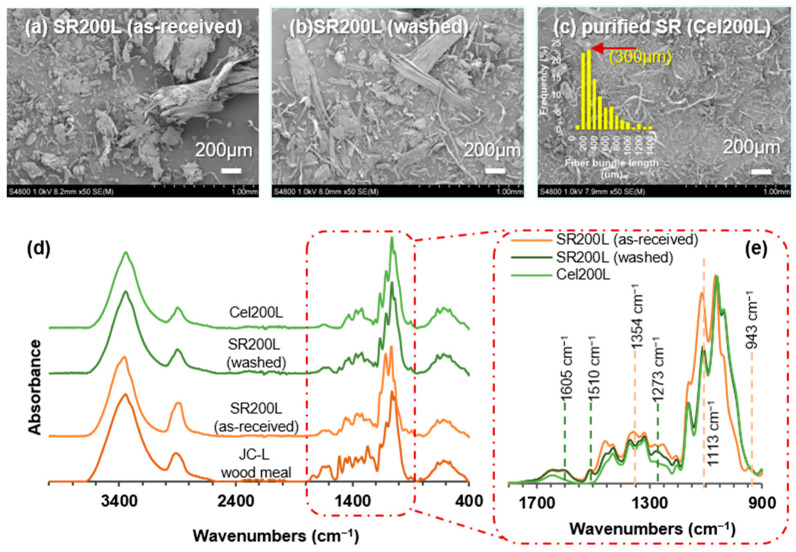
FE–SEM images of SR200L (**a**) as-received and (**b**) after washing to eliminate unreacted PEG200, (**c**) purified cellulose fiber bundles of Cel200L, and (**d**,**e**) purification process monitored by ATR-FTIR spectral analysis.

Thoroughly stirred purified cellulose slurries (2 wt%) were obtained under the same bleaching conditions, that is, 1 wt% acidified sodium chlorite (NaClO_2_/CH_3_COOH) at 75 °C for 2 h and 2 wt% potassium hydroxide solution at 80 °C for 1 h. For the sedimentation test, the slurries were left standing for 24 h and the distributions of their fiber bundles were then examined. The Cel200L sample, which exhibited the highest height ratio of sediment to suspension phase, was selected for CNC preparation. Panels (a), (b), and (c) of Figure 2 show field emission scanning electron microscopy (FE–SEM) images of the as-received SR200L, washed SR200L, and purified Cel200L, respectively. A high purity of well-distributed Cel200L (~80 wt%; Figure 2c) was obtained after the elimination of PEG and lignin from the as-received SR200L, a byproduct of GL200L production from JC-L and PEG200 (Figure 1). From optical micrographs, the estimated lengths of the fiber bundles in all samples were proximal to 300 μm (Figure 2c inset). The elimination of unreacted PEG and residual lignin was further confirmed in the attenuated total reflection Fourier transform infrared (ATR-FTIR) spectra of the purification process (Figure 2d). The characteristic peaks of PEG [7] at 1354 cm^−1^ (–CH_2_ wagging), 1113 cm^−1^ (C–O stretching), and 943 cm^−1^ (–CH_2_ rocking) appeared in the ATR-FTIR spectrum of the as-received SR200L but were undetected in the spectrum of washed SR200L (Figure 2e). After purification, the characteristic peaks of lignin at 1605 cm^−1^, 1510 cm^−1^, and 1273 cm^−1^ corresponding to skeletal aromatic ring vibrations and C=O stretching, skeletal ring vibrations, and G-ring breathing with C=O stretching [7], respectively, were also absent in the ATR-FTIR spectrum of Cel200L (Figure 2e).

### 2.2. Preparation Strategy and CNC Yields

**Figure 3:**
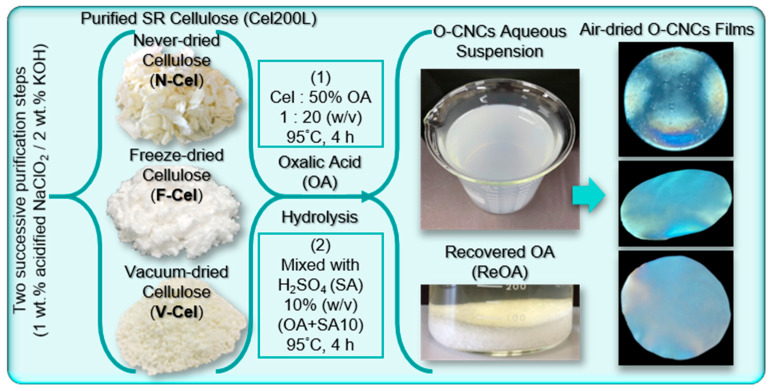
Schematic showing oxalic acid (OA) hydrolysis of different forms of cellulose from solid residues (SRs) in the softwood-derived glycol lignin (GL) production process. The right column displays cross-polarized photographs of the iridescent O-CNCs films.

O-CNCs were isolated from the Cel200L through acid hydrolysis using OA, a recyclable dicarboxylic acid that concurrently catalyzes the hydrolysis of amorphous cellulose segments and esterification, thereby introducing carboxylic acid functionality to isolated O-CNC surfaces [25]. As reported in several studies, the physical dimensions, surface charge, and the yield of O-CNCs largely depend on the source of starting cellulose materials, acid concentration, reaction temperature, reaction time, and the ratio of the cellulose fiber-to-acid solution [31,32,33,35]. In this study, the effect of the nature of the starting cellulose materials on the properties of the final O-CNCs was evaluated on different forms of purified Cel200L—the above-defined N-Cel, F-Cel, and V-Cel—under the same hydrolysis conditions (95 °C; 4 h; Cel200L to aqueous OA solution (50 wt%) of 1:20 (*w*/*v*) (see Figure 3)). The selected hydrolysis condition was based on the reported studies as shown in Table 1 [25,31,32,33] and the hydrolysis time (4 h) was determined through a preliminary trail on V-Cel for 2–6 h. The unreacted OA was recovered through OA crystallization from the hydrolysate (Figure 3). The performance of the recovered OA (ReOA) was evaluated over two cycles. The right column in Figure 3 displays cross-polarized photographs of the iridescent O-CNCs films resulting from the evaporation-induced self-assembly of an aqueous O-CNC suspension at room temperature. One of the unique properties of CNCs is their ability to form nematic or chiral nematic (cholesteric) liquid crystal phases at a certain CNC/water concentration. The appearance of iridescence revealed the photonic crystal character of the O-CNC films, implying their applicability to photonics and other specialty applications.

**Figure 4:**
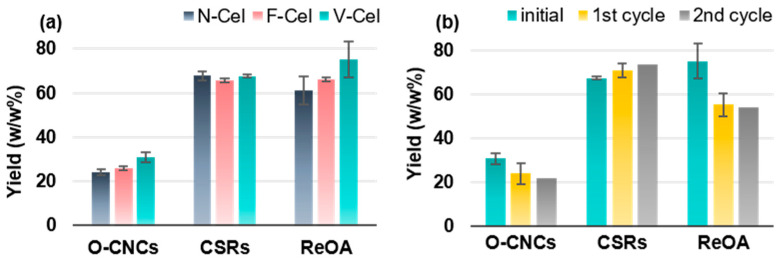
Yields of O-CNCs, remaining cellulosic solid residues (CSRs), and recovered oxalic acid (ReOA) from hydrolysis of (**a**) different forms of starting cellulose materials (N-Cel, F-Cel, and V-Cel) and (**b**) V-Cel using recycled oxalic acid (ReOA).

The yields of O-CNCs, cellulosic solid residues (CSRs), and recovered OAs (ReOAs) after N-Cel, F-Cel, and V-Cel hydrolysis are shown in Figure 4a. The isolation yields of O-CNCs ranged from 24% to 31% in the order of N-Cel < F-Cel < V-Cel (Table 1). The addition of 10 wt% sulfuric acid (SA10) notably increased the O-CNCs (N-Cel) yield (to 32%) but only marginally increased the yields of F-Cel and V-Cel (to ~2%). Collectively, drying (freeze-dried and vacuum-dried cellulose) affected the supermolecular structure of cellulose [34], thereby enhancing the acid hydrolysis and improving the O-CNCs yield from that of never-dried cellulose; nevertheless, the yield of O-CNCs derived from N-Cel improved after adding the cocatalyst (SA10).

Table 1 reports the O-CNC yields of the present study along with those of earlier studies [25,31,32,33]. At a fiber-to-acid solution (50 wt% OA) ratio of 1:10 (*w*/*w*) at 100 °C for 1 h [31], a maximum O-CNC yield of approximately 5% was obtained from all starting cellulose sources: hardwood bleached kraft eucalyptus pulp (BEP), macerated softwood spruce dissolving pulp (SDP), and cotton fiber [31]. Chen et al. [25] reported an O-CNC yield of ~16% from BEP hydrolyzed with 50 wt% OA at 100 °C for 4 h (fiber-to-acid solution ratio of 1:8, *w*/*v*).

In another study [32], the O-CNC yield from BEP increased from 9% to 25% and 34% after adding 10 wt% hydrochloric acid and 10 wt% SA, respectively. The hydrolysis was conducted at 90 °C for 4 h with a fiber-to-acid solution (40 wt% OA) ratio of 1:20 (*w*/*v*), close to the hydrolysis condition of the present study. The hydrolysis of hardwood BEP without the cocatalyst obtained obviously lower O-CNC yields (9% and 16%) than the hydrolysis of softwood JC-derived SRs in the present study (24–31%). Lin et al. [33] reported O-CNC yields of 24% and 33% from degreased cotton after 3 and 4 h hydrolysis, respectively, using 80 wt% OA (1:33 *w*/*w*) at 105 °C. The comparable O-CNC yield in the present study, even in concentrated acid (80 wt% OA) at an elevated temperature, was attributed to the different cellulose source and the insufficient hydrolysis of OA as a weak organic acid.

The non-CNC CSRs after O-CNC isolation were recovered through centrifugation and their yields were measured gravimetrically after freeze-drying. The cellulose loss was minimal after V-Cel hydrolysis (~2%) but slightly severe after N-Cel and F-Cel hydrolysis (~8%; see Figure 4a). Given the total O-CNC yield from V-Cel (~45%), the subsequent OA hydrolysis of the partially acid-hydrolyzed CSRs (V-Cel) for 2 h produced a further ~14% of O-CNCs. Furthermore, approximately 61–75% of OA was recovered (ReOA) by recrystallizing the hydrolysate (Figure 4a). Overall, V-Cel hydrolysis achieved a higher O-CNC yield with lower cellulose loss and a higher ReOA yield than N-Cel and F-Cel hydrolysis. Therefore, V-Cel was selected for the efficacy evaluation of ReOA. The O-CNC yields after the first- and second-recycled ReOA were 24% and 22%, respectively, which are lower than when using fresh OA (Figure 4b) but comparable with those of an earlier study using degreased cotton [33].

In addition, the sulfuric acid hydrolysis of V-Cel (V-Cel-to-SA solution (58 wt%) ratio of 1:20, *w*/*v*) at 55 °C for 30 and 60 min produced 52% and 60% of S-CNCs, respectively. Although the S-CNCs were obtained in higher yields than O-CNCs, the CSR recovery rate was low and 36% of the cellulose was lost. In a previous study [19], the S-CNC yield of hydrolyzed bleached softwood spruce pulp was 64% under the same hydrolysis condition (58 wt% SA at 55 °C for 30 min) combined with post homogenization treatment [19]. Wang et al. [32] isolated 35% of S-CNCs from BEP (58 wt% SA at 56 °C for 100 min). The CNC yield is known to depend on the cellulose source, hydrolysis conditions, and the pre- and post-treatment conditions. For all cellulosic sources under all hydrolytic conditions, the yields of O-CNCs (V-Cel) and S-CNCs (V-Cel) were comparable to or exceeded those of earlier studies [19,25,31,32,33].

### 2.3. Morphologies and Surface Charges

**Figure 5:**
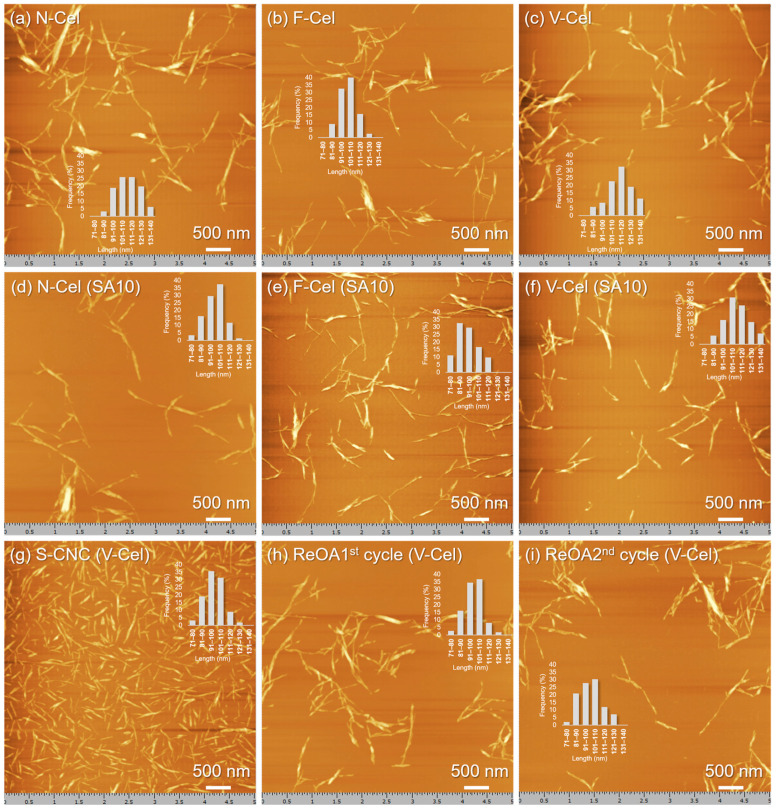
Topographic SPM images of the CNCs isolated from (**a**,**d**) N-Cel, (**b**,**e**) F-Cel, (**c**,**f**–**i**) V-Cel through oxalic acid (OA) hydrolysis alone (**a**–**c**), OA and 10 wt% sulfuric acid (SA10) hydrolysis (**d**–**f**), SA hydrolysis alone (**g**), and recycled OA (ReOA) hydrolysis (**h**,**i**). Insets show the length distributions of the corresponding CNCs (see Figure S1).

The morphology and nano-size observations of all CNCs were determined using scanning probe microscopy (SPM). Figure 5 presents the SPM images of CNCs produced from N-Cel, F-Cel, and V-Cel at a spatial resolution of 5 μm.

**Figure 6:**
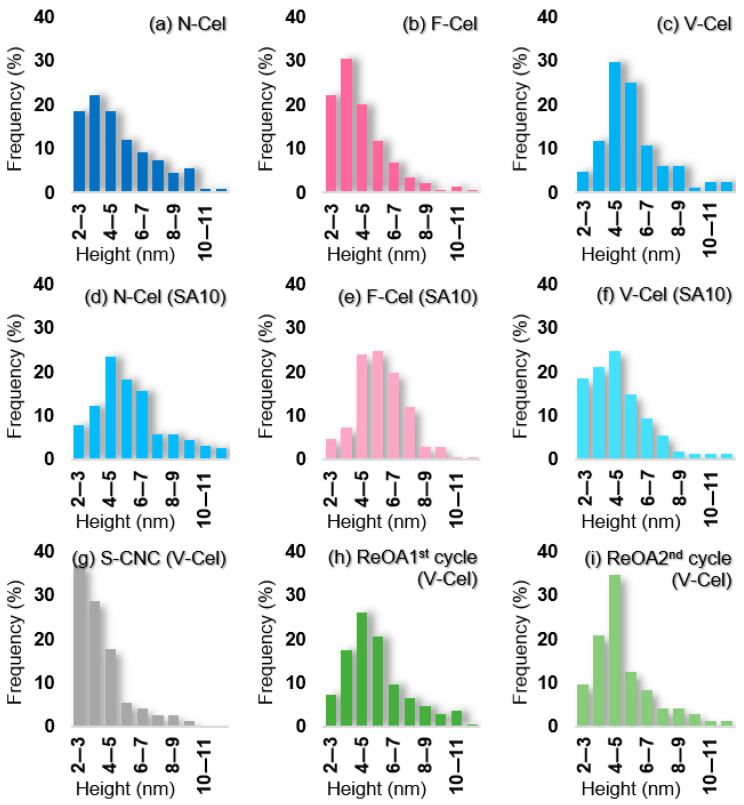
SPM-derived height (width) distributions of CNCs isolated from (**a**,**d**) N-Cel, (**b**,**e**) F-Cel, (**c**,**f**–**i**) V-Cel through oxalic acid (OA) hydrolysis alone (**a**–**c**), OA and 10 wt% sulfuric acid (SA10) hydrolysis (**d**–**f**), SA hydrolysis alone (**g**), and recycled OA (ReOA) hydrolysis (**h**,**i**).

O-CNCs were polydispersed and their nanoscale length and height (width) distributions were compared with those of S-CNCs (see Figure 5 and Figure 6). The average length of the O-CNCs ranged from 90 to 120 nm and the average width of separate particles ranged from 3 to 6 nm (8–20 nm in aggregates) (Table 2). The nature of the starting cellulose materials prepared from the same source (Cel200L) clearly influenced the physical dimensions of the O-CNCs. The O-CNCs prepared from F-Cel were shorter (103 ± 9 nm; Figure 5b) than those prepared from N-Cel (111 ± 12 nm; Figure 5a) and V-Cel (114 ± 13 nm; Figure 5c). Freeze-drying preserved the original fibrillar open network structure in F-Cel, providing an easily accessible surface structure for hydrolysis. Accordingly, F-Cel was hydrolyzed more quickly than N-Cel and V-Cel, leading to the constant breakdown of the disordered and even the crystalline domains under the same hydrolysis conditions. The addition of SA10 also appeared to slightly shorten the length (Figure 5d–f) from that of the O-CNCs prepared using OA alone (Figure 5a–c) but without altering the height (Figure 6, Table 2). Therefore, neither the hydrolytic reaction nor the surface esterification exerts an obvious thinning effect on the obtained O-CNCs. The O-CNC yields and the nanoscale length distributions are clearly related to the nature of the starting cellulose materials.

The average length distribution of the O-CNCs was 90–120 nm, which is shorter than those reported in earlier studies (174–450 nm) through the OA hydrolysis of softwood and/or hardwood kraft pulp [25,31,32,34]. This difference possibly reflects the different characteristics of the initial cellulose materials of the SRs obtained from GL production (softwood meal with an average size 1.6 mm) in this study and fresh pulp obtained from wood chip processing (commonly size: 25 × 25 × 4 mm^3^) [25,31,32,34].

The stability of the O-CNC suspensions is critical in nanocomposite material preparation. The dispersion stability of nanoscale particles can be evaluated through a zeta potential analysis; that is, by measuring the stable charge on the O-CNCs in an aqueous suspension (Table 2). The electrostatic repulsion between charged particles is believed to stabilize nanoscale particles in water. The zeta potentials of the O-CNCs isolated from N-Cel (−47.8 ± 0.7 mV), F-Cel (−54.0 ± 0.8 mV), and V-Cel (−49.5 ± 0.4 mV) confirmed the high physical stability of O-CNCs, conferred by the incorporation of negatively charged carboxylate half-ester groups on their surfaces. The surface charge was maximized on O-CNCs (F-Cel).

In addition, the surface charge on O-CNCs-SA10 prepared through the mixed-acid (OA + SA10) hydrolysis of F-Cel was very similar to that of O-CNCs (F-Cel), whereas mixed-acid hydrolysis slightly decreased and increased the surface charges on O-CNCs (N-Cel) and O-CNCs (V-Cel), respectively (Table 2). The zeta potential of O-CNCs (V-Cel) using once- and twice-recycled ReOA were similar to that of O-CNCs (V-Cel) using fresh OA. The determined values were also comparable to that of an earlier study (−48.1 ± 2 mV), in which macerated spruce SDP was hydrolyzed in 50 wt% OA [31]. Meanwhile, the zeta potential of S-CNCs were around −48.8 ± 0.7 mV owing to negatively charged ester sulfate groups incorporated onto the CNC surfaces. Furthermore, the zeta potential of the O-CNCs were positively correlated with the carboxyl group contents quantified by conductimetric titration (see Section 2.4).

### 2.4. Esterification and Carboxylation

During dicarboxylic acid (OA) hydrolysis, two concurrent acid-catalyzed reactions (the hydrolysis of amorphous cellulose segments and esterification) result in the incorporation of free carboxylic acid functionality onto CNC surfaces. It is hypothesized that only one carboxyl group participates in the oxalic acid esterification of cellulose; the other remains as a free carboxylic acid group imparting charge onto the CNC surfaces [25,36]. The esterification and carboxylation of CNC surfaces were evaluated through ATR-FTIR spectroscopy and conductimetric titration.

**Figure 7:**
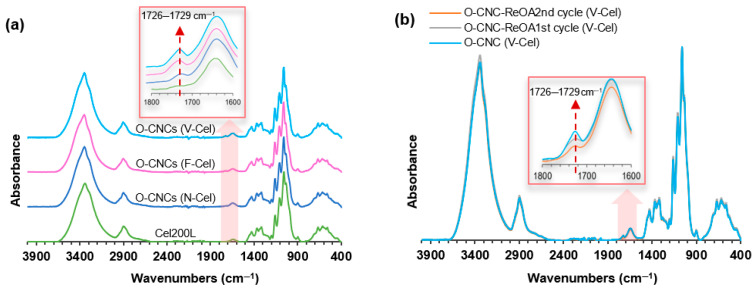
ATR-FTIR spectra of (**a**) O-CNCs prepared from OA hydrolysis of N-Cel, F-Cel, and V-Cel and (**b**) O-CNCs-ReOA using recycled OA. The spectra in (**a**,**b**) are compared with the spectra of Cel200L and O-CNCs isolated using fresh OA, respectively.

Similar to the ATR-FTIR spectrum of Cel200L, the spectra of O-CNC samples (Figure 7a) display the typical characteristic peaks of cellulose, suggesting that surface esterification through OA hydrolysis did not alter the nanocrystalline structure of cellulose. The strong absorption band around 3346 cm^−1^ and the absorption band at 2900–2902 cm^−1^ correspond to the stretching vibration of the hydroxyl (O–H) groups and symmetric C–H stretching vibrations, respectively. The absorbance at 1645 cm^−1^ is attributed to the –O–H bending vibrations of absorbed water, the sharp band at 1430 cm^−1^ arises from the symmetric bending vibrations of –CH_2_ groups, and the bands at 1367–1369 cm^−1^ and 1317 cm^−1^ belong to the –C–H bending vibrations in the cellulose I structure. The bands at 1160 cm^−1^ and 899–900 cm^−1^ correspond to the C–O–C antisymmetric stretching vibrations of the skeletal pyranose ring and the deformation of the β-glycosidc linkages in cellulose, respectively [25,31,35]. The new peak around 1726–1729 cm^−1^ in the O-CNC spectra, which is absent in the spectrum of Cel200L, corresponds to carbonyl (C=O) stretching vibrations, confirming that esterification introduces the carboxyl group to the O-CNC surfaces. The carbonyl stretching band is weak (Figure 7a inset), implying a moderate degree of esterification. In addition, the ATR-FTIR spectra of the O-CNCs-ReOA largely resembles that of O-CNCs isolated using fresh OA, although the degree of cellulose esterification reduces after the second cycle of ReOA (Figure 7b, inset).

As the signals of carboxylic acid (–COOH) and ester bonds (–COO–) overlap and cannot be easily distinguished, the content of carboxyl groups was quantified using conductimetric titration. The results are presented in Table 2. The carboxyl group content was higher in the O-CNCs prepared from F-Cel (0.22 mmol/g sample) than in O-CNCs (V-Cel) and O-CNCs (N-Cel) (0.15 and 0.13 mmol/g sample, respectively). The enhanced esterification in O-CNCs (F-Cel) was attributed to the preserved original fibrillar network with an easily accessible surface area after freeze-drying. After adding SA10, the carboxyl group contents of O-CNCs (F-Cel) and O-CNCs (V-Cel) slightly increased but that of O-CNCs (N-Cel) remained unchanged. These results imply that the nature of the starting cellulose materials also influences the reactivity of cellulose esterification. In addition, the cellulose esterification performance of ReOA reached that of fresh OA after the first cycle (0.15 mmol/g sample) but slightly decreased after the second cycle (0.11 mmol/g sample). As the samples were prepared from the same cellulose source under the same solvolysis conditions, their varying carboxyl group content clarifies that the nature of the starting cellulose material influences the degree of cellulose esterification.

### 2.5. Crystallinity

**Figure 8:**
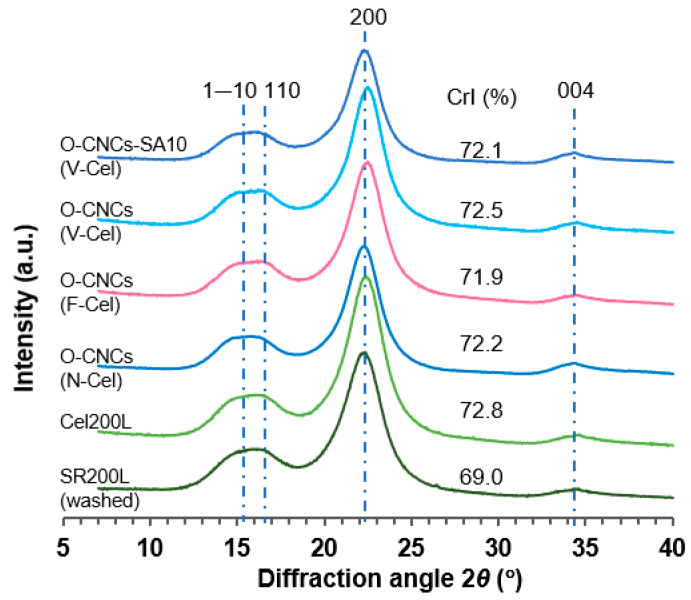
XRD patterns of the washed SR200L, purified Cel200L, and O-CNCs.

Figure 8 shows the X-ray diffraction (XRD) patterns of washed SR200L, purified Cel200L, and O-CNCs from N-Cel, F-Cel, and V-Cel recorded using the reflection method. The crystallinity index (CrI) was determined using the Segal method (Equation (2)) [37] (see Materials and Methods Section 3.4 for details).

The XRD patterns of all samples exhibited characteristic diffraction peaks around 2*θ* = 15.1°, 16.2°, 22.4°, and 34.5°assigned to the (1–10), (110), (200), and (004) reflection planes of cellulose I_β_, respectively. The crystallinity of Cel200L (72.8%) was enhanced after two successive purification treatments of SR200L (69.0%), which eliminated the residual lignin. As mentioned above, the raw material SR200L in the CNC preparation was a byproduct of the acid-catalyzed PEG solvolysis of softwood meal particles. The CrI of SR-derived Cel200L (72.8%) was comparable to that of bleached softwood kraft pulp fiber (73.4%) [28]. The XRD patterns and CrI (~72%) of O-CNCs were similar to that of Cel200L, indicating that OA hydrolysis did not disrupt the crystal structure of cellulose materials. Other studies using the Segal method have reported CrIs of 66.3% [32] in O-CNCs isolated from hardwood BEP using 40 wt% OA [32] and 81.3% [25] and 76.1% [31] in O-CNCs isolated from BEP using 50 wt% OA [25,31]. The CrI reached 85.7% in O-CNCs isolated through the 80 wt% OA hydrolysis of degreased cotton [33]. The hydrolysis conditions were 90 °C for 4 h in [32], 100 °C for 1.5 h in [25], 100 °C for 1 h in [31], and 105 °C for 4 h in [33]. The CrI values may depend on the source of starting cellulose materials (hardwood, softwood, and cotton) and the hydrolysis conditions. Nevertheless, the CrI values of the O-CNCs isolated from JC softwood residues in this study rank among the high crystallinity nanocellulose materials.

### 2.6. Thermal Stability

**Figure 9:**
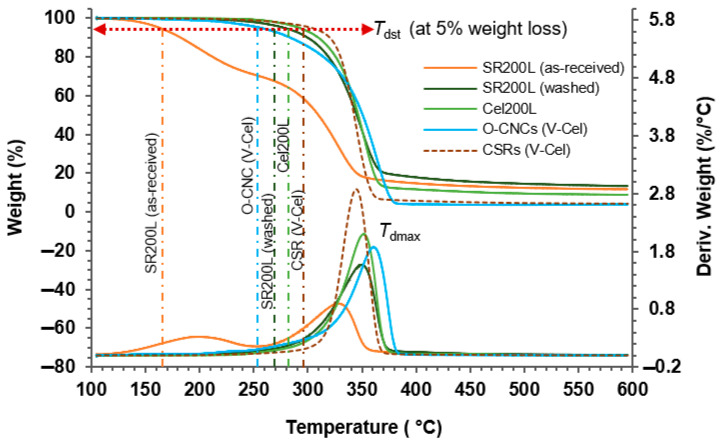
TGA/DTGA plots of as-received SR200L, washed SR200L, Cel200L, O-CNCs (V-Cel), and CSRs (V-Cel).

Thermogravimetric analyses (TGA) of the as-received SR200L, washed SR200L, Cel200L, and O-CNCs and their corresponding CSRs were conducted on a Q500TGA analyzer (TA Instruments-Water LLC, New Castle, DE, USA) under an N_2_ atmosphere. The thermal properties—the thermal decomposition starting temperature at 5% weight loss (*T*_dst_), maximum thermal decomposition temperature (*T*_dmax_), and charred residue content at 600 °C—are summarized in Table 3. Among these parameters, *T*_dst_ defines the thermal stability of the cellulosic materials. The transformation of TGA and differential TGA plots from as-received SR200L to O-CNCs (V-Cel) and CSRs (V-Cel) is presented in Figure 9.

As shown in Figure 9, the weight loss occurred between 150 °C and 400 °C as the cellulose decomposed. The small DTGA peak at 199 °C in the DTGA curve of the as-received SR200L is attributable to impregnated unreacted PEG200 and the impurities of the solvolysis reaction products, which lowered the *T*_dst_ to 163 °C. The *T*_dst_ increased from 278 °C after washing to 292 °C in purified Cel200L, which is the starting material of O-CNC preparation. The lower decomposition starting temperature in the O-CNC samples (253–282 °C; Table 3) than in Cel200L (292 °C) reflects the increased specific surface area of O-CNCs with nanoscale dimensions under heat exposure, along with the presence of active surface carboxyl groups [31,38]. Further supporting this postulation, the O-CNCs (F-Cel) with a higher carboxyl group content than O-CNCs (N-Cel) and O-CNCs (V-Cel) exhibited a lower *T*_dst_ than O-CNCs (N-Cel) and O-CNCs (V-Cel). Notably, O-CNCs-SA10 were more thermally stable than their corresponding O-CNCs. In addition, the *T*_dst_ of O-CNCs using twice-recycled ReOA (264 °C) was similar to that of O-CNCs using fresh OA (266 °C), suggesting the adequate efficacy of recycled ReOA. The *T*_dst_ of the O-CNCs (N-Cel) obviously exceeded those of O-CNCs (F-Cel) and O-CNCs (V-Cel), either with or without SA10. This result highlights that the form of the starting cellulose Cel200L (N-Cel, F-Cel, and V-Cel) influences the physical and thermal properties of the isolated O-CNCs.

The *T*_dst_ was followed by a pronounced weight loss in the 300–400 °C range, where the samples were thermally degraded by cellulose depolymerization, dehydration, and the decomposition of glycosyl units. Finally, only the charred residues remained. The O-CNCs exhibited higher *T*_dmax_ values (360–364 °C) than the starting Cel200L (352 °C). Accordingly, the charred residues of O-CNCs left fewer amounts at 600 °C (~3–4%) than Cel200L (~9%). In contrast, the *T*_dst_, *T*_dmax_, and charred residue content of S-CNCs (V-Cel) were 231 °C, 262 °C, and 18.6%, respectively. Obviously, the *T*_dst_ and *T*_dmax_ were approximately 30 °C and 100 °C lower, respectively, than those of O-CNCs (V-Cel).

The *T*_dst_ and *T*_dmax_ values of the corresponding CSRs were also evaluated. The CSRs exhibited higher *T*_dst_ values (278–306 °C) than their corresponding O-CNCs (253–282 °C). Since CSRs are partially acid-hydrolyzed cellulose materials with a high thermal stability, their potential range of applications is similar to that of microcrystalline cellulose.

## 3. Materials and Methods

### 3.1. Materials

The cellulose-rich solid residues (SRs) were obtained as a byproduct from the GL production process using softwood (Japanese Cedar, JC) biomass. Oxalic acid (98%, Wako special grade) as well as all other reagents such as sulfuric acid, acetic acid, sodium chlorite, potassium hydroxide, 0.1 M sodium hydroxide, and 0.1 M hydrochloric acid were of analytical grade and purchased from FUJIFILM Wako Pure Chemical Corporation, Osaka, Japan.

### 3.2. Purification of Cellulose from Solid Residue

The sampling size of approximately 150 g of filter-pressed cellulose-rich SRs was obtained from the GL production process through the acid-catalyzed PEG solvolysis of JC wood meals. The SRs were washed with distilled water to remove the remaining PEG (Figure 1). The washing step (15 min in 3 L distilled water with stirring) was performed three times and the resulting never-dried washed SRs were stored in the cold room (~10 °C). To estimate the yield of washed SRs after the removal of PEG, portions of the SRs were vacuum-dried for 2 d. A Klason lignin assay of washed SRs was performed as previously described [5]. The averages of duplicate assays were reported. The never-dried washed SRs (50 g dry weight basis) were bleached through two successive purification treatments: first with 1 wt% acidified sodium chlorite (NaClO_2_/CH_3_COOH) at 75 °C for 2 h; second with 2 wt% potassium hydroxide solution at 80 °C for 1 h. The cellulose slurry obtained after each purification step was vacuum-filtered and thoroughly washed with distilled water until the pH reached that of the washing water. The product was stored as never-dried purified cellulose (N-Cel) in the refrigerator.

### 3.3. Isolation of Cellulose Nanocrystals

The CNCs were prepared from purified cellulose Cel200L as shown in Figure 3. Some portions of the never-dried Cel200L (N-Cel) were freeze-dried (F-Cel) or vacuum-dried and milled (V-Cel). Based on a preliminary trial test, in which the hydrolysis time was varied from 2 to 6 h, the different forms of Cel200L (N-Cel, F-Cel, and V-Cel) were hydrolyzed with OA at 95 °C for 4 h. Briefly, 5 g of purified cellulose sample was added to the 100 mL of 50 wt% aqueous OA solution. The OA had been completely dissolved at 95 °C in advance and the reaction slurry was constantly stirred at 95 °C for 4 h. Each reaction was terminated by adding 200 mL of distilled water followed by two successive centrifugations at 3300 rpm for 5 min to remove the hydrolysate containing unreacted OA. This solution was kept in a cold room (~10 °C) overnight to precipitate the recrystallized OA (ReOA). The recovered ReOA was collected by vacuum filtration, rinsed with distilled water, and vacuum-dried for reuse in the next hydrolysis reaction.

The precipitated hydrolyzed cellulose was washed through centrifugation until the CNCs began to appear in the supernatant. The turbid supernatant CNC portion was then continuously collected through centrifugation (4500 rpm, 10 min). The resulting suspensions were dialyzed against distilled water in a dialysis tube (pore size of 5 Å, cutoff molecular weight, 14,000) until the pH reached that of distilled water and then transferred to an aqueous PEG20,000 (FUJIFILM Wako Pure Chemical Corporation, Osaka, Japan) bath to obtain a concentrated CNC sample. The resulting CNC samples, namely, the O-CNCs, were stored at 4 °C and/or freeze-dried for further analyses. The yield of the resulting O-CNCs was calculated by dividing the mass of O-CNCs by the mass of the starting cellulose materials.

For comparison, conventional SA hydrolysis of V-Cel was conducted in 58 wt% SA (V-Cel/acid ratio = 1:20, *w*/*v*) with stirring at 55 °C for 30 or 60 min [19]. The purified nanocrystal suspensions, namely, the S-CNCs were collected as described above and stored at 4 °C for further analyses.

### 3.4. Characterization of O-CNCs

The morphology of the freeze-dried SR200L (as-received and washed) and Cel200L was observed using field emission scanning electron microscopy (FE–SEM S-4800, Hitachi High-Tech Corp., Tokyo, Japan). The freeze-dried samples were loaded onto the carbon tape attached on the sample holder and coated with osmium plasma using a Neo osmium coater (Meiwafosis Co., Ltd., Tokyo, Japan). The FE–SEM images were recorded under high vacuum with an accelerating voltage of 1 kV.

The dimensions of the CNCs were estimated from SPM images obtained by an SPM9700HT (Shimadzu, Kyoto, Japan) equipped with a 10 μm scanner under ambient conditions. Dilute suspensions of the CNCs (0.01% *w*/*w*) were coated on a freshly cleaved mica surface with a spin coater (Opticoat MS-B100, Mikasa Co., Ltd., Osaka, Japan). A silicon tip (OMCL-AC160TS-C3, Olympus, Tokyo, Japan) with a nominal resonance frequency of ~300 kHz, a nominal spin constant of ~26 Nm^−1^, and a nominal tip radius of 7 nm was used to record the height and phase images over a 5 μm scan area using a phase mode. The resolution was 512 × 512 data points. For width determination, the height images were also analyzed using built-in image data processing software. The length distributions of CNCs were estimated from over 150 randomly chosen particles in each sample.

Attenuated total reflectance Fourier transform infrared spectroscopy (ATR-FTIR) spectra of the freeze-dried samples were obtained using a Nicolet is50 FTIR spectrometer (Thermo Fisher Scientific, Madison, WI, USA) equipped with a single-crystal diamond ATR top plate accessory. All spectra were recorded through 32 scans from 400 to 4000 cm^−1^ at 4 cm^−1^ resolution in absorbance mode.

The zeta potential was determined in 0.01% (*w*/*w*) aqueous O-CNC suspensions using a Nanoparticle analyzer SZ100 (Horiba Scientific, Kyoto, Japan). An appropriate amount of concentrated O-CNCs was diluted to 0.01% in ultrapure water. The dispersion was homogenized by ultrasonication for 30 sec in TAITEC Ultrasonic Homogenizer VP-300N equipped with a tapered microtip (VP-MT03, 6 mmΦ) (TAITEC Corporation, Saitama, Japan). The pH of all samples was between 6.5 and 7.4. The average values of triplicate measurements were reported.

The carboxyl group contents of the O-CNC suspensions were determined through conductimetric titration [39]. The concentrated O-CNCs (50 mg solid content) was suspended in 45 mL of distilled water and stirred for 1 h to obtain a well-dispersed suspension. After adding 15 mL of 0.01 M HCl, conductimetric titration was conducted with 0.04 M NaOH solution at 0.1 mL min^−1^ using a Metrohm 856 Conductivity Module (Herisau, Switzerland). The data were recorded by OMNIS Software 3.0.0. The carboxyl group content was calculated as:

(1) $$C=\frac{\left[NaOH\right]\times (V2-V1)}{w},$$

where *C* is the carboxyl group content (mmol/g sample), [NaOH] is the NaOH concentration (mol·L^−1^), *V*1 and *V*2 are the volume of NaOH (mL) at the equivalence point, and *w* is the solid weight of the O-CNC sample.

Wide-angle XRD patterns of selected samples were determined using a Rigaku SmartLab X-ray Diffractometer (Rigaku Corporation, Tokyo, Japan) equipped with nickel-filtered Cu-Kα radiation (λ = 0.1542 nm) at 40 kV and 50 mA. The freeze-dried samples were compacted into disk pellets under 2 tons using a KBr disk apparatus. The diffractograms were recorded over the 2*θ* range 5–40°at 0.5 min^−1^ using the SmartLab Studio II application software. The crystalline index (CrI) was calculated using the Segal method [37] as follows:

CrI (%) = (*I*_200_ − *I*_am_)/*I*_200_ × 100, (2)

where *I*_200_ is the total intensity of the (200) reflection peak (2*θ*: ~22.4°), and *I*_am_ is the amorphous intensity at 2*θ* = 18.5°.

The thermal behaviors of all samples were studied using a thermogravimetric analyzer (Q500TGA, TA Instruments-Water LLC, New Castle, DE, USA). A platinum pan (100 μL) loaded with a vacuum-dried or freeze-dried sample (7–8 mg) was initially heated to 105 °C at 10 °C min^−1^ and held at 105 °C for 20 min. Subsequently, it was heated to 850 °C at 10 °C min^−1^ under a nitrogen flow (60 mL min^−1^ in the sample compartment and 40 mL min^−1^ in the balance compartment). Duplicate datasets were analyzed using TA universal analysis 2000 software, where *T*_dst_ and *T*_dmax_ defined the temperatures at 5% weight loss and maximum weight loss, respectively. *T*_dst_ was recalculated once the weight stabilized at 105 °C.

## 4. Conclusions

In summary, the carboxylated O-CNCs from the same cellulose source with different physical forms (N-Cel, F-Cel, and N-Cel) were isolated under the same OA hydrolysis conditions. The different O-CNCs displayed varying physical dimensions, zeta potentials, carboxyl group contents, and thermal stabilities but maintained the original crystal structure of cellulose. Clearly, freeze-drying and vacuum-drying altered the physical and structural properties of never-dried cellulose to some extent, thereby affecting the characteristics of the obtained O-CNCs. Although the hydrolysis conditions were not optimized to maximize the O-CNC yield, the present results are comparable with those of earlier studies using different cellulosic sources under various OA hydrolysis conditions. The present results clarify the importance of the physical and structural properties of the starting cellulose material in CNC preparation. The SR byproduct must be effectively utilized along with GLs to realize the full potential of the current GL production process in future biorefineries. The present study provides a foundation for the advancement of GL production technology.

## Figures and Tables

**Table 1:** Reported yields of O-CNCs prepared from wood pulp and cotton.

Cellulose Source	Sample Condition	Oxalic Acid Concentration (wt%)	Solid: Liquid Ratio	Temp. (°C)	Time (h)	Yield (%)	Carboxyl Group Content (mmol/g)	Reference
Bleached SRs- softwood Japanese cedar (200–500 μm)	(1) N-Cel (2) F-Cel (3) V-Cel	50	1:20 (*w*/*v*)	95	4	(1) 24.3 (2) 25.9 (3) 30.9	(1) 0.13 (2) 0.22 (3) 0.15	this study
Bleached kraft eucalyptus pulp (BEP)	soaked in water disintegration 2% consistency air-dried	30–70 50 50 70	1:8 (*w*/*v*)	80–120 100 100 100	0.5–4 4 1.5 1	1.4–24.7 15.8 11.0 24.7	0.11–0.39 NA 0.17 0.23	[25]
(1) BEP (2) Macerated spruce dissolving pulp (SDP) (3) Native cotton fiber (QFP)	soaked in water disintegration 2% consistency air-dried	(1) 10–50 50 (2) 10–50 50 (3) 10–50 50	1:10 (*w*/*v*)	100	1	(1) 0–4.4 4.4 (2) 0–5.0 5.0 (3) 0–5.8 5.8	0.20 0.24 0.07	[31]
BEP	not mentioned	40 40 + SA10 ^a^ 40 + HA10 ^b^	1:20 (*w*/*v*)	90	4	9.2 33.9 25.2	0.11 0.42 0.32	[32]
Degreased cotton (20–50 μm)	dried fiber	60–80 60 80	1:33 (*w*/*w*)	100–110 100 105	2–4 3 4	14.7–33.4 14.7 33.4	0.11–0.17 0.12 0.11	[33]

^a^ SA10 = 10 wt% sulfuric acid, ^b^ HA10 = 10 wt% hydrochloric acid.

**Table 2:** Characteristics of O-CNCs and S-CNCs.

CNCs	Length ^a^	Height ^b^	Zeta Potential	Carboxyl Group
	(nm)	(nm)	(mV)	mmol/g CNCs
O-CNC (N-Cel)	111 ± 12	5 ± 2	−47.8 ± 0.7	0.13
O-CNC-SA10 (N-Cel)	101 ± 10	6 ± 2	−43.8 ± 0.4	0.13
O-CNC (F-Cel)	103 ± 9	4 ± 2	−54.0 ± 0.8	0.22
O-CNC-SA10 (F-Cel)	93 ± 12	6 ± 2	−54.5 ± 0.5	0.23
O-CNC (V-Cel)	114 ± 13	5 ± 2	−49.5 ± 0.4	0.15
O-CNC-SA10 (V-Cel)	110 ± 10	5 ± 2	−51.2 ± 0.6	0.23
O-CNC-ReOA1st cycle (V-Cel)	100 ± 10	6 ± 2	−47.6 ± 0.6	0.15
O-CNC-ReOA2nd cycle (V-Cel)	100 ± 12	5 ± 2	−47.0 ± 0.5	0.11
S-CNCs (V-Cel)	99 ± 10	4 ± 2	−48.8 ± 0.7	-

^a^,^b^ Estimated from scanning probe microscopy images, represented as histograms in ^a^ Figure 5 (insets), also see in ^a^ Figure S1 and ^b^ Figure 6.

**Table 3:** Thermal properties of as-received SR200L, washed SR200L, Cel200L, O-CNCs, and corresponding CSRs.

	*T*_dst_	*T*_dmax_	Char Residues	*T*_dst_	*T*_dmax_	Char Residues
	(°C)	(°C)	(%)	(°C)	(°C)	(%)
SR200L (as-received)	163	328	11.7	-	-	-
SR200L (washed)	278	350	13.4	-	-	-
Cel200L	292	352	8.9	-	-	-
O-CNCs				CSRs		
N-Cel	271	360	3.4	279	337	2.3
F-Cel	253	360	3.2	306	352	5.3
V-Cel	257	364	3.9	304	345	4.3
O-CNCs-SA10				CSRs (SA10)		
N-Cel	282	362	3.3	278	358	2.8
F-Cel	258	360	4.2	306	343	4.3
V-Cel	266	364	2.7	304	338	3.7
O-CNCs-ReOA				CSRs (ReOA)		
V-Cel-ReOA1st cycle	258	361	3.1	304	343	4.2
V-Cel-ReOA2nd cycle	264	360	3.5	-	-	-
S-CNCs	231	262	18.6	-	-	-

*T*_dst_—thermal decomposition starting temperature at 5% weight loss. *T*_dmax_—maximum thermal decomposition temperature.

## Data Availability

The original contributions presented in this study are included in the article. Further inquires can be directed to the corresponding authors.

## Supplementary Materials

The following supporting information can be downloaded at: https://www.mdpi.com/article/10.3390/molecules30142922/s1, Figure S1. SPM-derived length distributions of CNCs isolated from (a,d) N-Cel, (b,e) F-Cel, (c,f–i) V-Cel through oxalic acid (OA) hydrolysis alone (a–c), OA and 10 wt% sulfuric acid (SA10) hydrolysis (d–f), SA hydrolysis alone (g), and recycled OA (ReOA) hydrolysis (h,i). (Enlarged histograms of “insets” displayed in Figure 5).
