# Supplementary material for: Characterization of Carboxylated Cellulose Nanocrystals Isolated Through Oxalic Acid Hydrolysis from Solid Residues of Softwood-Derived Glycol Lignin Production
Source: Molecules. 2025 Jul 10;30(14):2922. doi: 10.3390/molecules30142922 (PMC12299898; doi:10.3390/molecules30142922)
Supplement: Supplementary file 1 [file molecules-30-02922-s001.zip › molecules-3673408-supplementary.pdf]

Supplementary Materials

Characterization of Carboxylated Cellulose Nanocrystals Isolated through Oxalic Acid Hydrolysis from Solid Residues of Softwood-Derived Glycol Lignin Production

List of Contents

Figure S1. SPM-derived length distributions of CNCs isolated from (a,d) N-Cel, (b,e) F-Cel, (c,f,–i) V-Cel through oxalic acid (OA) hydrolysis alone (a–c), OA and 10 wt% sulfuric acid (SA10) hydrolysis (d–f), SA hydrolysis alone (g), and recycled OA (ReOA) hydrolysis (h,i).

Number of pages: 2

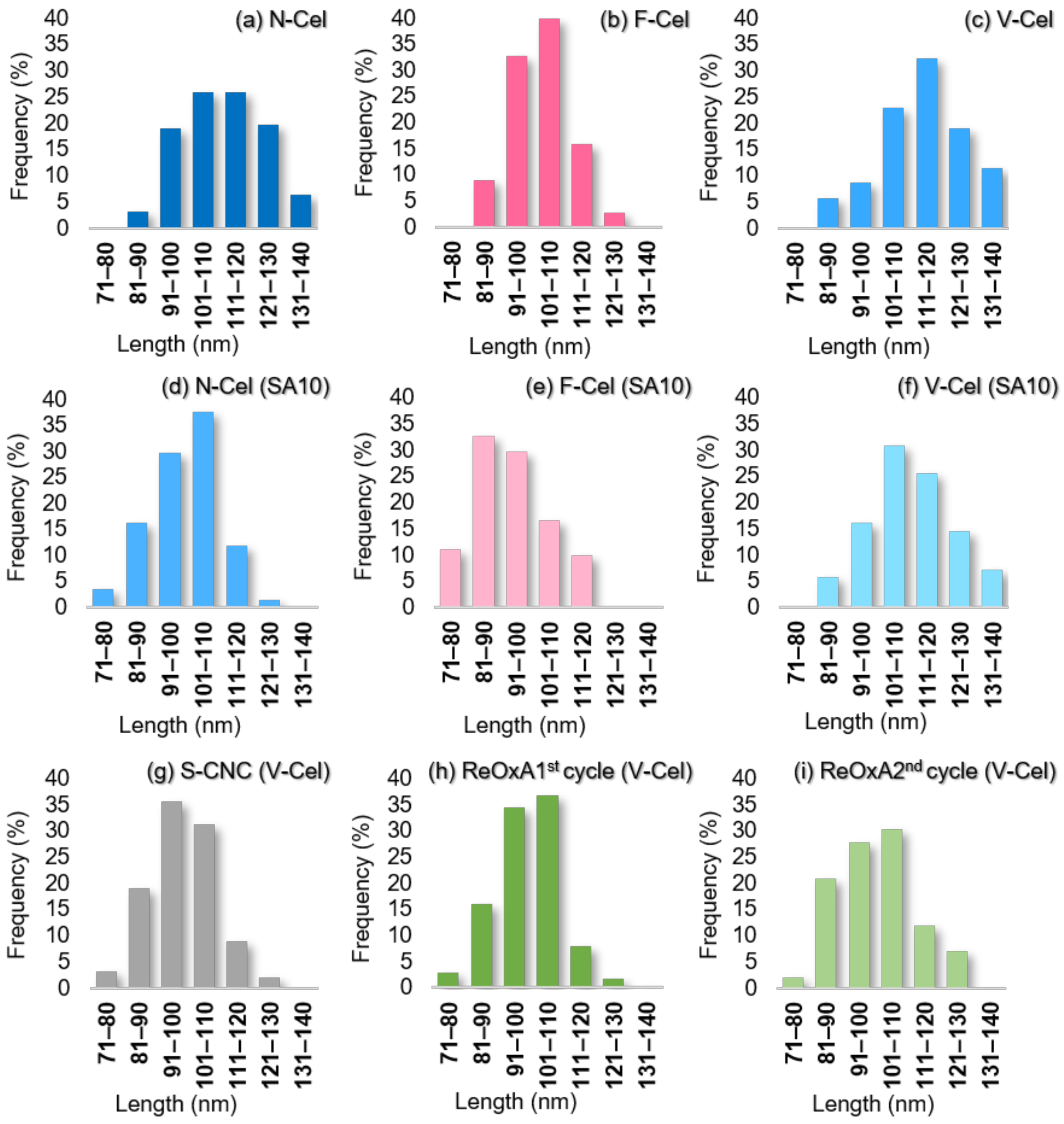

Figure S1. SPM-derived length distributions of CNCs isolated from (a,d) N-Cel, (b,e) F-Cel, (c,f–i) V-Cel through oxalic acid (OA) hydrolysis alone (a–c), OA and 10 wt% sulfuric acid (SA10) hydrolysis (d–f), SA hydrolysis alone (g), and recycled OA (ReOA) hydrolysis (h,i). (Enlarged histograms of "insets" displayed in Figure 5)
